# 4-Eth­oxy-3-meth­oxy­benzaldehyde

**DOI:** 10.1107/S160053681302761X

**Published:** 2013-11-06

**Authors:** Zorica Leka, Sladjana B. Novaković, Goran A. Bogdanović, Jovana Muškinja, Rastko D. Vukićević

**Affiliations:** a Faculty of Metallurgy and Technology, University of Montenegro, Cetinjski put bb, 81000 Podgorica, Montenegro; b Vinča Institute of Nuclear Sciences, Laboratory of Theoretical Physics and Condensed Matter Physics, PO Box 522, University of Belgrade, 11001 Belgrade, Serbia; c Faculty of Sciences, Department of Chemistry, University of Kragujevac, R. Domanovića 12, 34000 Kragujevac, Serbia

## Abstract

In the title compound, C_10_H_12_O_3_, all non-H atoms are approximately coplanar, with an r.m.s. deviation of 0.046 Å. In the crystal, very weak C—H⋯O inter­actions link the mol­ecules into sheets parallel to (101).

## Related literature

For the bioactivity of de­hydro­zingerone derivatives and their role in the synthesis of heterocycles, see: Tatsuzaki *et al.* (2006 ▶); Kubra *et al.* (2013 ▶); Panda & Chowdary (2008 ▶); Mostahar *et al.* (2007 ▶). For related crystal structures, see: Matos Beja *et al.* (1997 ▶); Velavan *et al.* (1995 ▶).
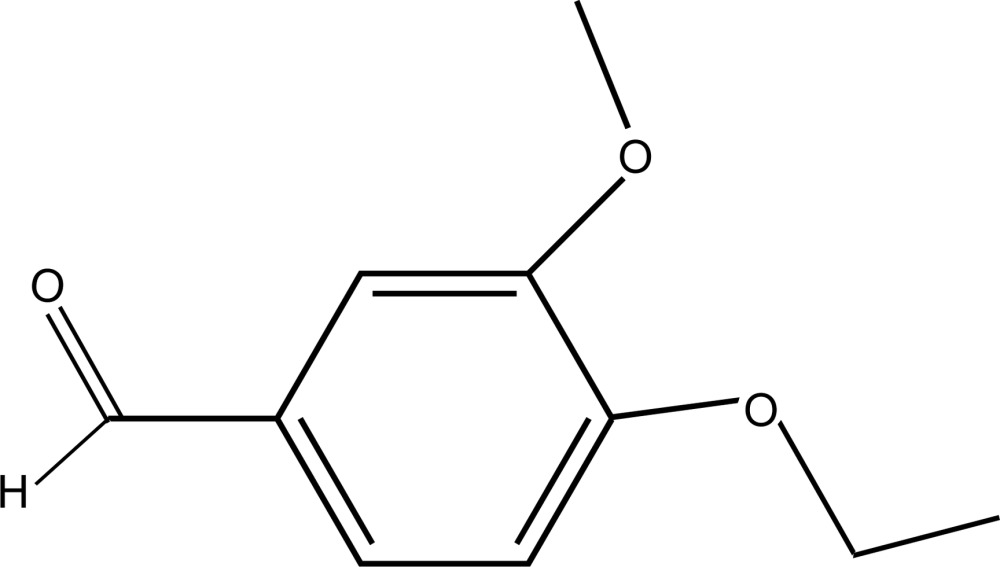


## Experimental

### 

#### Crystal data

C_10_H_12_O_3_
*M* *_r_* = 180.20
Monoclinic, 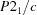

*a* = 11.5314 (16) Å
*b* = 8.7905 (11) Å
*c* = 9.3363 (13) Å
β = 97.339 (14)°
*V* = 938.6 (2) Å^3^
*Z* = 4
Cu *K*α radiation
μ = 0.78 mm^−1^
*T* = 293 K
0.39 × 0.17 × 0.14 mm

#### Data collection

Agilent Gemini S diffractometer
Absorption correction: multi-scan (*CrysAlis PRO*; Agilent, 2013 ▶) *T*_min_ = 0.933, *T*_max_ = 1.000
6035 measured reflections
1848 independent reflections
1299 reflections with *I* > 2σ(*I*)
*R*_int_ = 0.021

#### Refinement

*R*[*F* ^2^ > 2σ(*F* ^2^)] = 0.047
*wR*(*F* ^2^) = 0.148
*S* = 1.04
1848 reflections
120 parameters
H-atom parameters constrained
Δρ_max_ = 0.12 e Å^−3^
Δρ_min_ = −0.17 e Å^−3^

### 

Data collection: *CrysAlis PRO* (Agilent, 2013 ▶); cell refinement: *CrysAlis PRO*; data reduction: *CrysAlis PRO*; program(s) used to solve structure: *SHELXS97* (Sheldrick, 2008 ▶); program(s) used to refine structure: *SHELXL97* (Sheldrick, 2008 ▶); molecular graphics: *ORTEP-3 for Windows* (Farrugia, 2012 ▶) and *Mercury* (Macrae *et al.*, 2006 ▶); software used to prepare material for publication: *WinGX* (Farrugia, 2012 ▶), *PLATON* (Spek, 2009 ▶) and *PARST* (Nardelli, 1995 ▶).

## Supplementary Material

Crystal structure: contains datablock(s) I, global. DOI: 10.1107/S160053681302761X/zq2209sup1.cif

Structure factors: contains datablock(s) I. DOI: 10.1107/S160053681302761X/zq2209Isup2.hkl

Supplementary material file. DOI: 10.1107/S160053681302761X/zq2209Isup3.cml

Additional supplementary materials:  crystallographic information; 3D view; checkCIF report

## Figures and Tables

**Table 1:** Hydrogen-bond geometry (Å, °)

*D*—H⋯*A*	*D*—H	H⋯*A*	*D*⋯*A*	*D*—H⋯*A*
C1—H1⋯O1^i^	0.93	2.67	3.547 (3)	157
C10—H10*B*⋯O2^ii^	0.96	2.62	3.525 (2)	156

Symmetry codes: (i) 
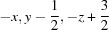
; (ii) 
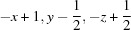
.

**Fig. 1.**
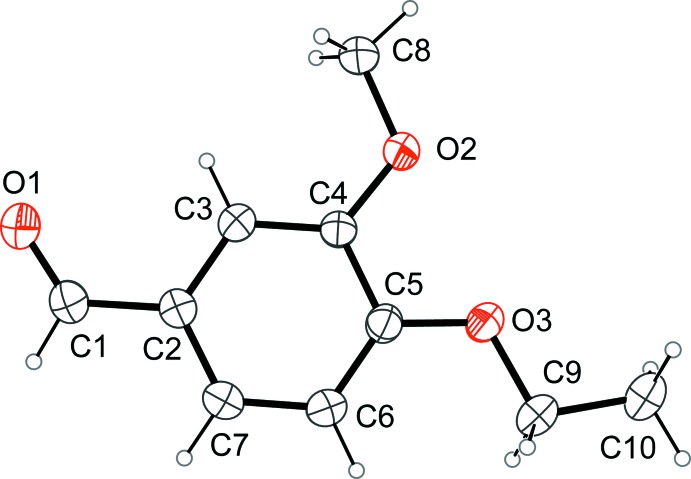
The molecular structure of the title compound, with atom labels and 30% probability displacement ellipsoids for non-H atoms.

**Fig. 2.**
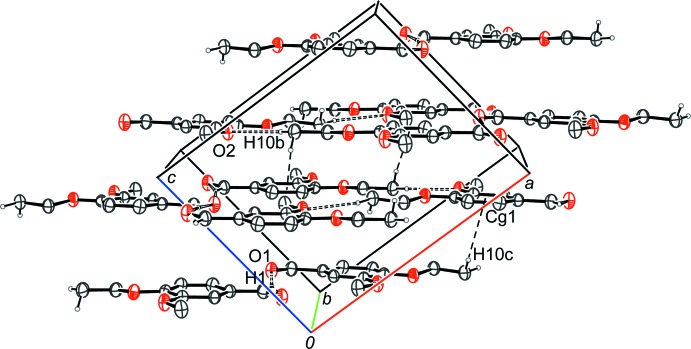
Three-dimensional layered structure of the title compound. All H atoms which are not involved in intermolecular interactions are omitted for clarity.

## supplementary crystallographic information

### 1. Comment

Dehydrozingerone derivatives belong to an important class of compounds, not only due to their different bioactivities (Tatsuzaki *et al.*, 2006; Kubra *et al.*, 2013), but also as the key substrates in synthesis of some heterocycles (Panda *et al.*, 2008), particularly flavones (Mostahar *et al.*, 2007). They can be synthesized by condensation of the corresponding aromatic aldehyde with acetone. Thus, starting substrate in synthesis of ethyl derivative of dehydrozingerone is 4-ethoxy-3-methoxybenzaldehyde (I), compound obtained by simple methylation of vanillin.

In the title structure, all non-hydrogen atoms are approximately coplanar with a mean deviation of 0.046 Å (Fig. 1). A somewhat higher displacement of 0.102 (2) Å has been observed for atom C9 belonging to ethoxy moiety. The dihedral angle between the best planes through the phenyl ring and the non-H atoms of ethoxy moiety is 8.1 (1)°. The aromatic C—C bond lengths are in the expected range of 1.368 (2)–1.411 (2) Å (Table 2). The five C—O bonds have various lengths. The shortest length is found for the carbonyl C1—O1 = 1.204 (2) bond in accordance with the prevaling double bond character.

The crystal structure exhibits no conventional hydrogen bonding. The molecules are held together by weak C–H···O and van der Waals interactions. The two C—H···O intermolecular contacts shorter than the sum of the van der Waals radii [C1—H1 = 0.93; H1···O1= 2.67 Å; C1—H1···O1^i^ = 157.3° and C10—H10*B* = 0.96, H10*B*···O2 = 2.62 Å, C10—H10*B*···O2^ii^ = 156.4° (symmetry codes: i = -*x*,+*y* - 1/2,-*z* + 3/2; ii = -*x* + 1,+*y* - 1/2,-*z* + 1/2)] connect the molecules into a sheet parallel to (101). These sheets further connect into three-dimensional structure by C—H···π interaction [C10—H10*c* = 0.96, H10*c*···C*g*1 = 3.00 Å C10—H10*c*···C*g*1^iii^ = 147° [(symmetry code: iii = -*x* + 1,-*y*,-*z* + 1)] (Fig. 2). The approximate distance between the adjacent parallel sheets is 3.5 Å. For related crystal structures, see Matos Beja *et al.* (1997) and Velavan *et al.* (1995).

### 2. Experimental

Diethyl sulfate was dropped into a water solution of sodium hydroxide and vanillin. The reaction mixture was stirred overnight at 50°C and cooled at room temperature resulting firstly an oily product, which on standing gave crude crystal 4-ethoxy-3-methoxybenzaldehyde.

One gram of crude 4-ethoxy-3-methoxybenzaldehyde was stirred vigorously in 150 ml of boiling water, the hot mixture filtered of through a cotton pad. The obtained milky-white emulsion upon overnight cooling at room temperature gave crystal needles of 4-ethoxy-3-methoxybenzaldehyde.

### 3. Refinement

All H atoms were included in calculated positions and treated as riding with *d*(C—H) equal to: 0.96 Å (CH_3_), 0.97 Å (CH_2_) or 0.93 Å (aromatic CH) and *U*_iso_(H) equal to: 1.5 *U*_eq_(C) for CH_3_ or 1.2 *U*_eq_(C) for CH_2_ and CH.

### Crystal data

**Table:**

C_10_H_12_O_3_	*F*(000) = 384
*M**_r_* = 180.20	*D*_x_ = 1.275 Mg m^−^^3^
Monoclinic, *P*2_1_/*c*	Cu *K*α radiation, λ = 1.54180 Å
Hall symbol: -P 2ybc	Cell parameters from 1676 reflections
*a* = 11.5314 (16) Å	θ = 3.9–71.1°
*b* = 8.7905 (11) Å	µ = 0.78 mm^−^^1^
*c* = 9.3363 (13) Å	*T* = 293 K
β = 97.339 (14)°	Needle, white
*V* = 938.6 (2) Å^3^	0.39 × 0.17 × 0.14 mm
*Z* = 4	

### Data collection

**Table:**

Agilent Gemini S diffractometer	1848 independent reflections
Radiation source: Enhance (Cu) X-ray Source	1299 reflections with *I* > 2σ(*I*)
Graphite monochromator	*R*_int_ = 0.021
Detector resolution: 16.3280 pixels mm^-1^	θ_max_ = 73.2°, θ_min_ = 3.9°
ω scans	*h* = −14→14
Absorption correction: multi-scan (*CrysAlis PRO*; Agilent, 2013)	*k* = −10→8
*T*_min_ = 0.933, *T*_max_ = 1.000	*l* = −11→9
6035 measured reflections	

### Refinement

**Table:**

Refinement on *F*^2^	Primary atom site location: structure-invariant direct methods
Least-squares matrix: full	Secondary atom site location: difference Fourier map
*R*[*F*^2^ > 2σ(*F*^2^)] = 0.047	Hydrogen site location: inferred from neighbouring sites
*wR*(*F*^2^) = 0.148	H-atom parameters constrained
*S* = 1.04	*w* = 1/[σ^2^(*F*_o_^2^) + (0.0746*P*)^2^ + 0.0716*P*] where *P* = (*F*_o_^2^ + 2*F*_c_^2^)/3
1848 reflections	(Δ/σ)_max_ < 0.001
120 parameters	Δρ_max_ = 0.12 e Å^−^^3^
0 restraints	Δρ_min_ = −0.17 e Å^−^^3^

### Special details

**Table:**

Experimental. Absorption correction: empirical absorption correction using spherical harmonics, implemented in SCALE3 ABSPACK scaling algorithm (CrysAlis PRO; Agilent, 2013)

### Fractional atomic coordinates and isotropic or equivalent isotropic displacement parameters (Å^2^)

**Table:**

	*x*	*y*	*z*	*U*_iso_*/*U*_eq_	
O1	−0.01326 (13)	−0.00997 (18)	0.78722 (16)	0.0954 (5)	
O2	0.27667 (11)	0.22381 (13)	0.46226 (14)	0.0818 (4)	
O3	0.37383 (11)	−0.00811 (13)	0.35924 (13)	0.0780 (4)	
C1	0.03443 (17)	−0.1075 (2)	0.7257 (2)	0.0824 (5)	
H1	0.0130	−0.2076	0.7411	0.099*	
C2	0.12260 (15)	−0.0833 (2)	0.62934 (18)	0.0683 (5)	
C3	0.15663 (15)	0.0635 (2)	0.59500 (18)	0.0675 (5)	
H3	0.1230	0.1470	0.6348	0.081*	
C4	0.23881 (14)	0.08576 (18)	0.50352 (17)	0.0642 (4)	
C5	0.29161 (15)	−0.0414 (2)	0.44602 (18)	0.0665 (5)	
C6	0.25783 (15)	−0.1856 (2)	0.47925 (19)	0.0768 (5)	
H6	0.2918	−0.2696	0.4406	0.092*	
C7	0.17310 (17)	−0.2063 (2)	0.5703 (2)	0.0793 (6)	
H7	0.1502	−0.3043	0.5917	0.095*	
C8	0.22711 (16)	0.3550 (2)	0.5194 (2)	0.0889 (6)	
H8A	0.1438	0.3535	0.4938	0.133*	
H8B	0.2588	0.4447	0.4804	0.133*	
H8C	0.2453	0.3554	0.6227	0.133*	
C9	0.43966 (16)	−0.1315 (2)	0.3099 (2)	0.0812 (6)	
H9A	0.3872	−0.2051	0.2583	0.097*	
H9B	0.4838	−0.1823	0.3916	0.097*	
C10	0.52047 (17)	−0.0694 (3)	0.2129 (2)	0.0930 (7)	
H10A	0.4761	−0.0211	0.1314	0.140*	
H10B	0.5659	−0.1507	0.1799	0.140*	
H10C	0.5717	0.0037	0.2645	0.140*	

### Atomic displacement parameters (Å^2^)

**Table:**

	*U*^11^	*U*^22^	*U*^33^	*U*^12^	*U*^13^	*U*^23^
O1	0.0975 (10)	0.0929 (10)	0.1040 (11)	−0.0069 (8)	0.0448 (9)	−0.0036 (8)
O2	0.0900 (8)	0.0605 (7)	0.1034 (9)	0.0055 (6)	0.0452 (7)	0.0059 (6)
O3	0.0846 (8)	0.0715 (8)	0.0838 (8)	0.0123 (6)	0.0332 (7)	0.0019 (6)
C1	0.0844 (12)	0.0782 (12)	0.0887 (13)	−0.0103 (10)	0.0265 (10)	0.0006 (10)
C2	0.0694 (10)	0.0658 (11)	0.0714 (10)	−0.0022 (8)	0.0152 (8)	0.0024 (8)
C3	0.0679 (10)	0.0652 (10)	0.0715 (10)	0.0047 (8)	0.0170 (8)	−0.0022 (8)
C4	0.0661 (9)	0.0578 (10)	0.0706 (10)	0.0041 (7)	0.0162 (8)	0.0045 (7)
C5	0.0675 (9)	0.0694 (11)	0.0643 (9)	0.0050 (8)	0.0153 (8)	0.0017 (8)
C6	0.0872 (12)	0.0633 (11)	0.0834 (12)	0.0063 (9)	0.0239 (10)	−0.0036 (9)
C7	0.0920 (13)	0.0600 (11)	0.0887 (12)	−0.0049 (9)	0.0224 (10)	0.0028 (9)
C8	0.0965 (14)	0.0606 (11)	0.1171 (16)	0.0027 (9)	0.0431 (12)	−0.0010 (10)
C9	0.0803 (12)	0.0827 (12)	0.0839 (12)	0.0139 (10)	0.0235 (10)	−0.0118 (10)
C10	0.0837 (13)	0.1090 (17)	0.0911 (14)	0.0149 (11)	0.0294 (11)	−0.0087 (12)

### Geometric parameters (Å, º)

**Table:**

O1—C1	1.204 (2)	C6—C7	1.387 (2)
O2—C4	1.3618 (18)	C6—H6	0.9300
O2—C8	1.421 (2)	C7—H7	0.9300
O3—C5	1.355 (2)	C8—H8A	0.9600
O3—C9	1.433 (2)	C8—H8B	0.9600
C1—C2	1.457 (2)	C8—H8C	0.9600
C1—H1	0.9300	C9—C10	1.484 (3)
C2—C7	1.376 (2)	C9—H9A	0.9700
C2—C3	1.398 (2)	C9—H9B	0.9700
C3—C4	1.368 (2)	C10—H10A	0.9600
C3—H3	0.9300	C10—H10B	0.9600
C4—C5	1.411 (2)	C10—H10C	0.9600
C5—C6	1.374 (2)		
			
C4—O2—C8	117.27 (13)	C2—C7—H7	119.7
C5—O3—C9	117.93 (14)	C6—C7—H7	119.7
O1—C1—C2	126.0 (2)	O2—C8—H8A	109.5
O1—C1—H1	117.0	O2—C8—H8B	109.5
C2—C1—H1	117.0	H8A—C8—H8B	109.5
C7—C2—C3	119.20 (16)	O2—C8—H8C	109.5
C7—C2—C1	119.78 (17)	H8A—C8—H8C	109.5
C3—C2—C1	121.02 (17)	H8B—C8—H8C	109.5
C4—C3—C2	120.83 (16)	O3—C9—C10	108.51 (16)
C4—C3—H3	119.6	O3—C9—H9A	110.0
C2—C3—H3	119.6	C10—C9—H9A	110.0
O2—C4—C3	125.22 (15)	O3—C9—H9B	110.0
O2—C4—C5	115.38 (14)	C10—C9—H9B	110.0
C3—C4—C5	119.40 (15)	H9A—C9—H9B	108.4
O3—C5—C6	125.06 (16)	C9—C10—H10A	109.5
O3—C5—C4	115.17 (15)	C9—C10—H10B	109.5
C6—C5—C4	119.77 (16)	H10A—C10—H10B	109.5
C5—C6—C7	120.13 (17)	C9—C10—H10C	109.5
C5—C6—H6	119.9	H10A—C10—H10C	109.5
C7—C6—H6	119.9	H10B—C10—H10C	109.5
C2—C7—C6	120.65 (17)		
			
O1—C1—C2—C7	177.57 (19)	O2—C4—C5—O3	0.8 (2)
O1—C1—C2—C3	−2.7 (3)	C3—C4—C5—O3	−178.53 (14)
C7—C2—C3—C4	0.2 (3)	O2—C4—C5—C6	−178.87 (16)
C1—C2—C3—C4	−179.46 (15)	C3—C4—C5—C6	1.7 (3)
C8—O2—C4—C3	0.3 (3)	O3—C5—C6—C7	179.56 (16)
C8—O2—C4—C5	−179.05 (16)	C4—C5—C6—C7	−0.8 (3)
C2—C3—C4—O2	179.20 (15)	C3—C2—C7—C6	0.8 (3)
C2—C3—C4—C5	−1.5 (3)	C1—C2—C7—C6	−179.51 (17)
C9—O3—C5—C6	−6.9 (3)	C5—C6—C7—C2	−0.5 (3)
C9—O3—C5—C4	173.41 (14)	C5—O3—C9—C10	177.85 (15)

### Hydrogen-bond geometry (Å, º)

**Table:**

*D*—H···*A*	*D*—H	H···*A*	*D*···*A*	*D*—H···*A*
C1—H1···O1^i^	0.93	2.67	3.547 (3)	157
C10—H10*B*···O2^ii^	0.96	2.62	3.525 (2)	156

Symmetry codes: (i) −*x*, *y*−1/2, −*z*+3/2; (ii) −*x*+1, *y*−1/2, −*z*+1/2.
